# Contributions of MyD88-dependent receptors and CD11c-positive cells to corneal epithelial barrier function against *Pseudomonas aeruginosa*

**DOI:** 10.1038/s41598-017-14243-w

**Published:** 2017-10-23

**Authors:** Matteo M. E. Metruccio, Connie Tam, David J. Evans, Anna L. Xie, Michael E. Stern, Suzanne M. J. Fleiszig

**Affiliations:** 1 School of Optometry, University of California, Berkeley, CA 94720 USA; 2 College of Pharmacy, Touro University California, Vallejo, CA 94592 USA; 3 ImmunEyz, Laguna Niguel, CA 92677 USA; 4 Graduate Groups in Vision Science, Microbiology, and Infectious Diseases & Immunity, University of California, Berkeley, CA 94720 USA; 5 Present Address: Cole Eye Institute, Cleveland Clinic, Cleveland, OH 44195 USA

## Abstract

Previously we reported that corneal epithelial barrier function against *Pseudomonas aeruginosa* was MyD88-dependent. Here, we explored contributions of MyD88-dependent receptors using vital mouse eyes and confocal imaging. Uninjured IL-1R (−/−) or TLR4 (−/−) corneas, but not TLR2 (−/−), TLR5 (−/−), TLR7 (−/−), or TLR9 (−/−), were more susceptible to *P. aeruginosa* adhesion than wild-type (3.8-fold, 3.6-fold respectively). Bacteria adherent to the corneas of IL-1R (−/−) or TLR5 (−/−) mice penetrated beyond the epithelial surface only if the cornea was superficially-injured. Bone marrow chimeras showed that bone marrow-derived cells contributed to IL-1R-dependent barrier function. *In vivo*, but not *ex vivo*, stromal CD11c+ cells responded to bacterial challenge even when corneas were uninjured. These cells extended processes toward the epithelial surface, and co-localized with adherent bacteria in superficially-injured corneas. While CD11c+ cell depletion reduced IL-6, IL-1β, CXCL1, CXCL2 and CXCL10 transcriptional responses to bacteria, and increased susceptibility to bacterial adhesion (>3-fold), the epithelium remained resistant to bacterial penetration. IL-1R (−/−) corneas also showed down-regulation of IL-6 and CXCL1 genes with and without bacterial challenge. These data show complex roles for TLR4, TLR5, IL-1R and CD11c+ cells in constitutive epithelial barrier function against *P. aeruginosa*, with det*a*ils dependent upon *in vivo* conditions.

## Introduction

The healthy cornea is extremely resistant to bacterial infection, largely because its multilayered epithelial surface provides a barrier to microbes^1^. While contact lens wear does not overtly injure the epithelium, it can render the cornea susceptible to infection by opportunistic pathogens such as *Pseudomonas aeruginosa*, resulting in sight-threatening keratitis^2^. Understanding how lens wear alters the corneal epithelial barrier to allow microbial access requires foundational knowledge about how the barrier is maintained when a lens is not worn.

Our previous studies, and work done by others in the field, over the past three decades has identified multiple effectors of corneal epithelial barrier function. They include previously known and novel antimicrobial peptides, surface-expressed and soluble mucins, surfactant proteins, and junctional complexes^3–7^. Much less is known about how these defenses are regulated, the importance of which is illustrated by the fact that corneal epithelial cells differ vastly in vulnerability to inoculated bacteria depending on whether they are grown *in vitro* or *in vivo* ^4^. *In vitro*, even small inocula of *P. aeruginosa* quickly kill/invade corneal epithelial cells grown in culture^8^. *In vivo*, healthy corneal epithelium resists adhesion by extremely high inocula (e.g. ~10^11^ CFU/mL)^9^, and can continue to resist bacterial penetration even when superficial injury enables bacterial adhesion. Shedding light on why corneal epithelial cells are more resistant *in vivo*, we previously found that tear (mucosal) fluid modulates corneal epithelial defenses^10^. Not yet known, however, is how corneal cells sense and respond to *in vivo* factors that modulate epithelial barrier function.

The regulation of epithelial barrier function (during health) *in vivo* has in general received very little attention in the literature, with studies primarily focused on the regulation of inflammatory and immune responses during infection (disease), assisted by the availability of infection models. Animal models for studying opportunistic pathogens generally enable susceptibility by bypassing epithelial barriers. For example, corneal infection is studied using either a scratching method to derail the epithelial barrier, or microbes are injected across it into the underlying stroma wherein the disease process is initiated^11–14^. Studying maintenance of health in the face of bacterial challenge, which is the usual outcome, requires different animal models and a separate tool-kit of outcome measures.

We previously developed a suite of imaging technologies that enable 3D and temporal subcellular localization and quantification of bacterial distribution within corneas without tissue processing or even dissection of the cornea from the eyeball^9^. Using those methods, we showed that corneal epithelial barrier function against *Pseudomonas aeruginosa* adhesion and subsequent penetration, required MyD88^9^, an adaptor molecule required for most TLR- and IL-1R- mediated signaling cascades^15^. This result was somewhat surprising considering that MyD88-dependent signaling is generally thought to trigger inflammation and other events during disease, as opposed to being involved in constitutive maintenance of health. Knowing whether the same, or different, MyD88-dependent receptors and signaling events as those regulating inflammation are also involved in MyD88-dependent epithelial barrier function will be important for developing related therapies to combat inflammation or infection.

Here, we tested the hypothesis that one or more TLRs and/or the IL-1R, was required for corneal epithelial barrier function during health. We also examined the relative contributions of resident corneal and bone marrow-derived cells given that both cell types can express MyD88-dependent receptors^16,17^. The results showed that multiple MyD88-dependent receptors, and both cell types, can contribute to corneal epithelial barrier function during health, with relative roles depending on the integrity of the superficial epithelial cells, and whether or not the eye is studied *in vivo*.

## Results

### TLR4, TLR5 and the IL-1R each contribute to the epithelial barrier against *P. aeruginosa*

Since we have shown that corneal epithelial barrier function against *P. aeruginosa* was MyD88-dependent^9^, and given that MyD88 is an adaptor for TLR and IL-1R signaling, we investigated the contributions of TLRs and the IL-1R to corneal defense against *P. aeruginosa* during health. Wild-type and gene-knockout mouse eyes were challenged with *P. aeruginosa* and imaged *ex vivo* as previously described^9^.

**Figure 1:**
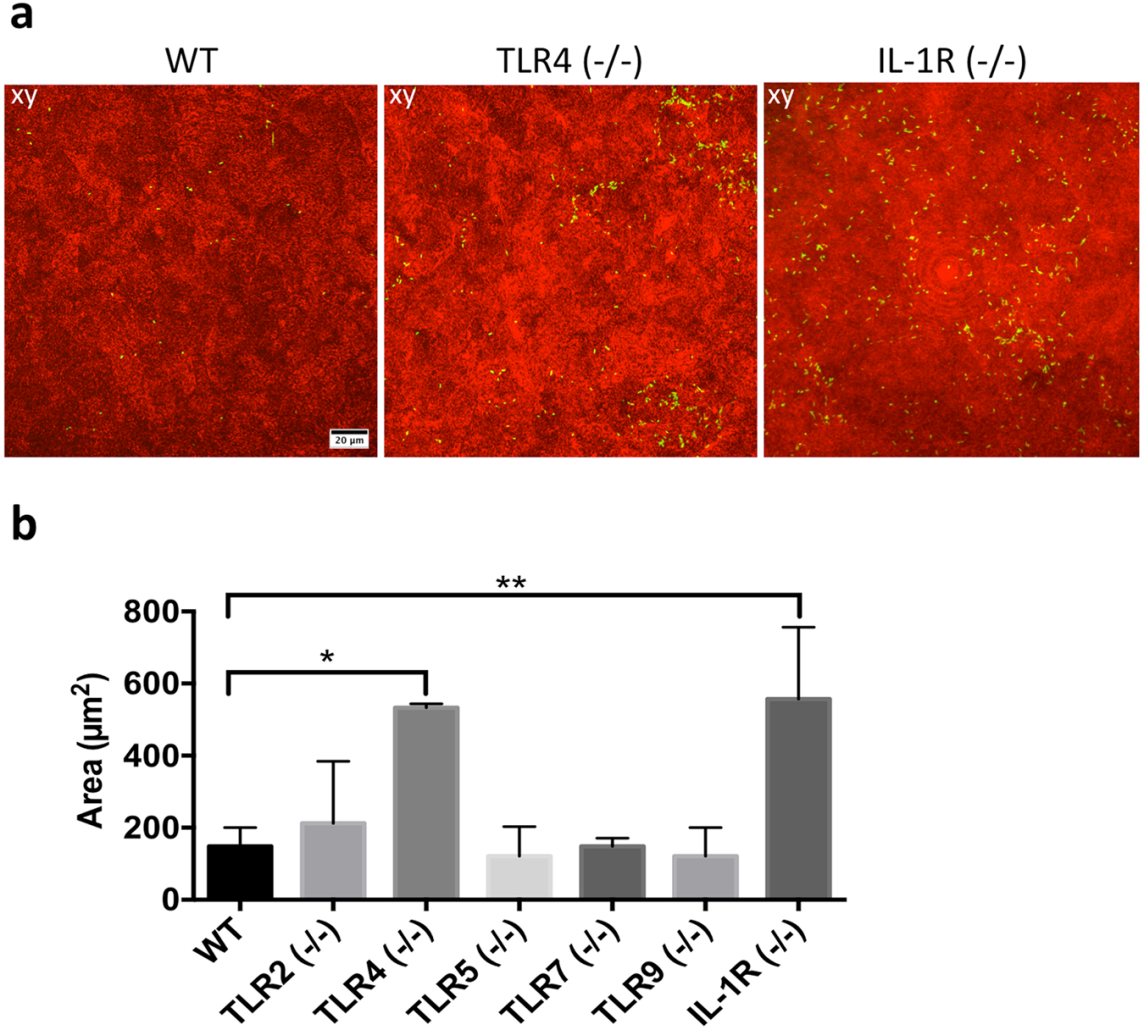
In healthy corneas, TLR4 and IL-1R contribute to barrier function against *P. aeruginosa* adhesion in an *ex vivo* model. Murine eyeballs were washed in PBS, placed in ~10^11^ CFU/mL PAO1-GFP for 6 h at 35 °C, rinsed with PBS then imaged by confocal microscopy. (**a**) Corneal images show increased bacterial adhesion in TLR4 (−/−) and IL-1R (−/−) versus wild-type (WT) eyes in healthy (non-blotted) eyes. Panels xy represent maximum intensity projections of the z dimension, generated using ImageJ. The corneal epithelium is shown in red (reflection) and bacteria are green (GFP). (**b**) Quantification of PAO1 adhesion (see Methods) in WT, TLR2 (−/−), TLR4 (−/−), TLR5 (−/−), TLR7 (−/−), TLR9 (−/−) and IL-1R (−/−) healthy corneas from 4 or more fields per eye, and three biological replicates. *p < 0.05, **p < 0.01, Kruskal-Wallis with Dunn’s multiple comparison test. 60x objective.

When healthy eyes were used, i.e. freshly excised, both IL-1R (−/−) and TLR4 (−/−) corneas showed increased bacterial adhesion compared to wild-type (Fig. 1a) with 3.8-fold and 3.6-fold increases respectively (Fig. 1b). Despite increased adhesion, bacteria did not penetrate beyond the surface (data not shown). Significant differences in bacterial adhesion were not observed between wild-type and TLR2 (−/−), TLR5 (−/−), TLR7 (−/−) and TLR9 (−/−) eyes (Fig. 1b).

**Figure 2:**
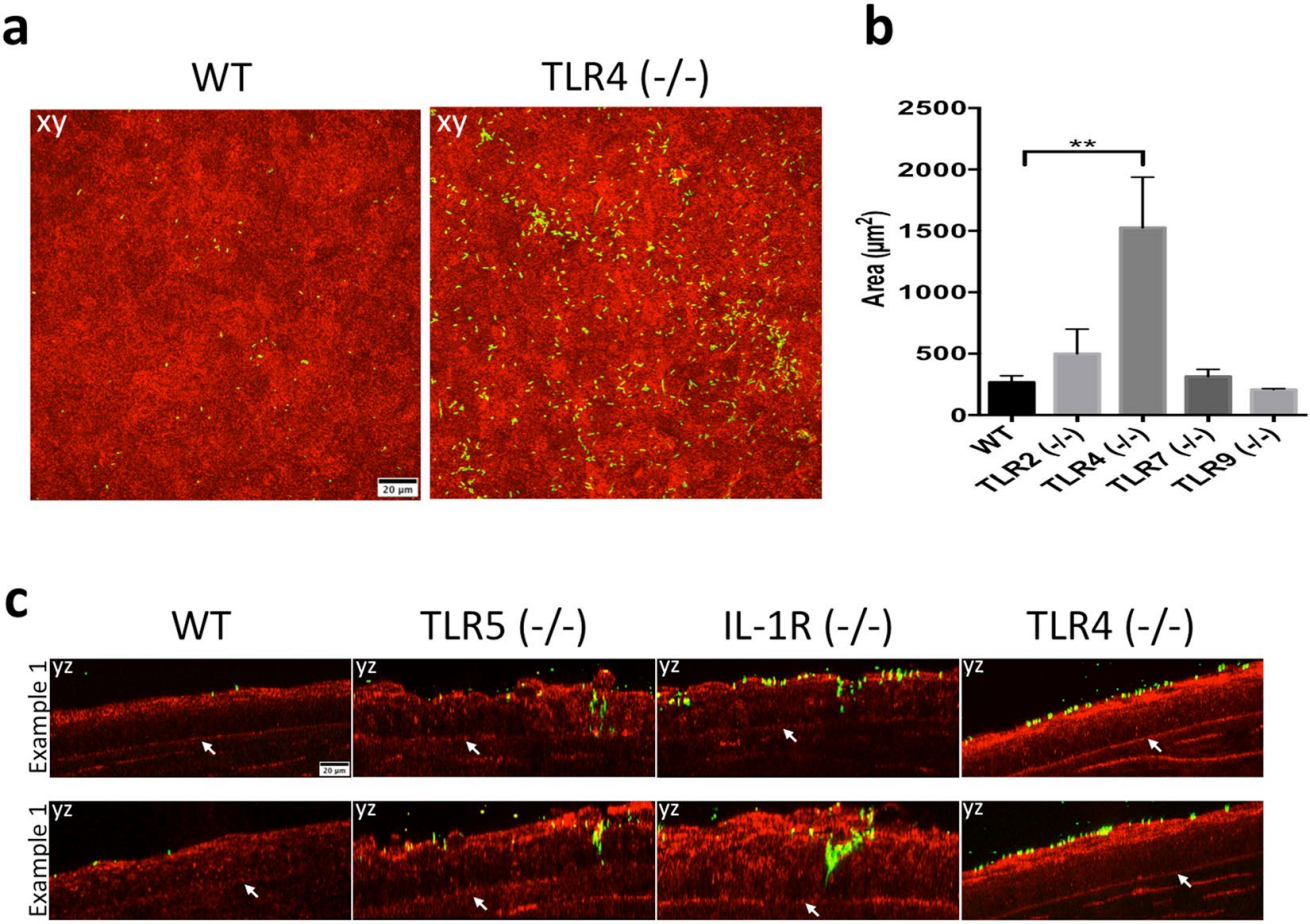
In superficially-injured (blotted) corneas TLR4 contributes to corneal defense against *P. aeruginosa* adhesion, but not epithelial penetration, and both IL-1R and TLR5 protect against bacterial penetration in an *ex vivo* model. (**a**) Corneal images of blotted WT and TLR4 (−/−) corneas showing greatly increased adhesion by PAO1-GFP. (**b**) Quantification of bacterial adhesion to superficially-injured (blotted) corneas from 4 or more fields per eye and three biological replicates showing > 5-fold increase in corneal adhesion in TLR4 (−/−) eyes. **p < 0.01 versus WT, Kruskal-Wallis with Dunn’s multiple comparison test. (**c**) *P. aeruginosa* epithelial penetration of blotted corneas in TLR5 (−/−) and IL-1R (−/−) eyes, but not in WT or TLR4 (−/−). Panels xy are maximum intensity projections. Panel yz represents optical sections (yz orthoslices, 2 µm thick) of representative areas in the field showing bacterial penetration (white arrows indicate the basal lamina). Corneal epithelium shown in red (reflection) and bacteria are green (GFP). 60x objective.

Eyes were then superficially-injured before bacterial inoculation, a procedure that involves blotting the epithelial surface with tissue paper enabling increased bacterial adhesion and fluorescein staining in wild-type mice^9^. Blotted TLR4 (−/−) corneas showed 5.7-fold increased bacterial adhesion compared to blotted wild-type corneas (Fig. 2a and b). However, blotted corneas of TLR2 (−/−), TLR7 (−/−), or TLR9 (−/−) mice did not show significant differences in bacterial adhesion compared to wild-type (Fig. 2b). Previously, we showed that tissue paper blotted murine corneas did not allow *P. aeruginosa* to penetrate the epithelium (despite increased adhesion) unless corneas were also pretreated with EGTA, or mice were deficient in Surfactant Protein D^6^. In the present study, however, bacteria were able to penetrate the epithelium of blotted TLR5 (−/−) and IL-1R (−/−) corneas, but not that of blotted wild-type or TLR4 (−/−) corneas (Fig. 2c). *P. aeruginosa* did not penetrate blotted corneal epithelium of TLR2 (−/−), TLR7 (−/−), or TLR9 (−/−) eyes (data not shown).

### IL-1R expression in both bone marrow-derived and non-bone marrow-derived cells contributes to epithelial barrier function against *P. aeruginosa* adhesion

**Figure 3:**
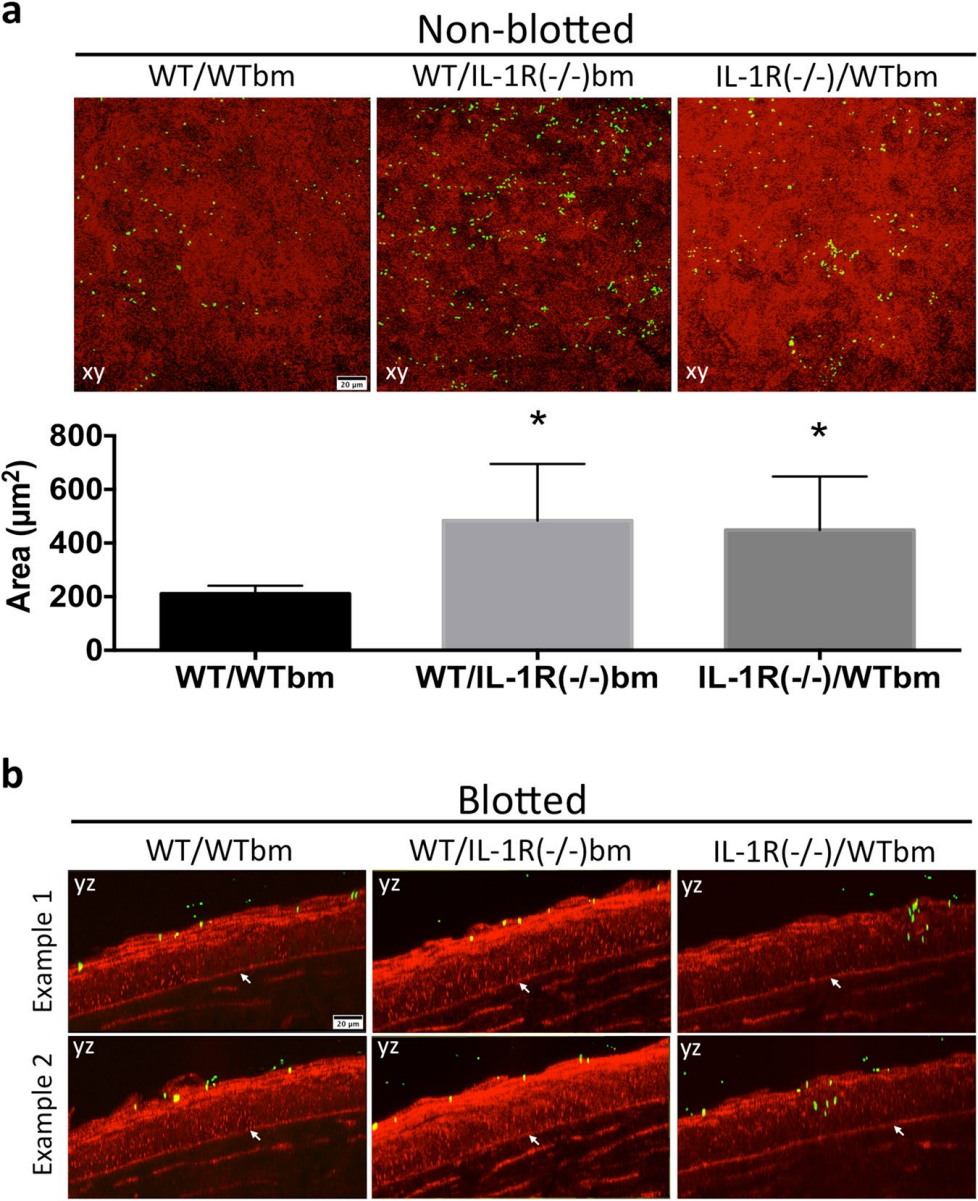
(**a**) Corneal images and quantification of *P. aeruginosa* PAO1 adhesion to uninjured corneas of bone marrow chimeric mice after bacterial challenge for 6 h *ex vivo*. WT mice complemented with IL-1R (−/−) bone marrow (bm)-derived cells [WT/IL-1R (−/−) bm], and IL-1R (−/−) mice complemented with WT bone marrow-derived cells [IL-1R (−/−)/WTbm] each showed increased bacterial adhesion compared to WT mice complemented with WT bone marrow-derived cells [WT/WTbm]. Quantification of adherent bacteria was determined from 4 or more fields per eye, and three biological replicates, *p < 0.05, Kruskal-Wallis with Dunn’s multiple comparison test. (**b**) Corneal images of superficially-injured bone-marrow chimeric mice showing no bacterial penetration of the epithelium after 6 h in WT mice complemented with IL-1R (−/−) bone marrow-derived cells, but bacteria did penetrate IL-1R (−/−) corneas complemented with WT bone marrow-derived cells [IL-1R (−/−)/WTbm]. Panels xy are maximum intensity projections. Panels yz represent optical sections (yz orthoslices, 2 µm thick) of areas in the field showing bacterial penetration (white arrows indicate the basal lamina). The corneal epithelium is red (reflection), bacteria are green (GFP). 60x objective.

Beside epithelial cells and nerves, the murine cornea harbors a diverse population of myeloid cells, mostly macrophages and dendritic cells^18–20^, some present even when the cornea is healthy. Since IL-1R contributed to defenses against bacterial adhesion and penetration, and since both bone marrow-derived cells and epithelial cells express this receptor, we next explored the relative roles of bone marrow and non-bone marrow-derived cells in mediating these defenses. Chimeric mice were generated harboring either a WT bone marrow lineage in an IL-1R (−/−) background, or an IL-1R (−/−) bone marrow in a WT background. Eyes were challenged with *P. aeruginosa* for 6 h *ex vivo* then imaged. Figure 3a shows that the transfer of bone marrow-derived cells from WT mice into the IL-1R (−/−) background partially rescued the increase in bacterial adhesion to IL-1R (−/−) corneas seen previously [mean ( ± standard deviation) of 2.5 (±0.4)-fold increase in Fig. 3a versus a mean of 3.8 (±0.2)-fold in Fig. 1b, p < 0.05, Student’s t-Test]. Similarly, transfer of IL-1R (−/−) bone marrow-derived cells into a WT background also increased bacterial adhesion, although again to a lower extent compared to IL-1R (−/−) corneas [mean of 2.6 (±0.2)-fold increase in Fig. 3a versus a mean of 3.8 (±0.2)-fold in Fig. 1b, p < 0.01, Student’s t-Test]. Bacteria did not penetrate the epithelium of any of the non-blotted chimeric mice despite bacterial adhesion (data not shown). These data suggest that the role of IL-1R in preventing *P. aeruginosa* adhesion to healthy corneas likely involves both epithelial and bone marrow-derived cells, or a cross-talk between them, each contributing to approximately half of the increased adhesion observed in IL-1R (−/−) eyes.

### Non-bone marrow-derived cells are the main contributors for IL-1R mediated barrier function against *P. aeruginosa* penetration after superficial epithelial injury

Given the contribution of both bone marrow-derived and non-bone marrow-derived cells to IL-1R-dependent barrier function against *P. aeruginosa* adhesion to the corneal epithelium, we tested if the same was true for bacterial penetration after superficial injury. In blotted corneas, bacterial penetration was observed in IL-1R (−/−) mice complemented with WT bone marrow-derived cells, but not in other chimeras, after 6 h challenge with *P. aeruginosa ex vivo* (Fig. 3b), indicating that IL-1R expressed in the bone marrow-derived cells was not sufficient to block the penetration phenotype previously observed in blotted IL-1R (−/−) eyes (Fig. 2c). Complementation of WT mice with IL-1R (−/−) bone marrow-derived cells did not allow bacterial penetration (Fig. 3b). In conclusion, IL-1R expressed in the corneal epithelium (or other non-bone marrow derived cells) appeared to be responsible for preventing *P. aeruginosa* penetration through the corneal epithelium in blotted eyes.

### Corneal CD11c+ cells respond to *P. aeruginosa* challenge *in vivo*

**Figure 4:**
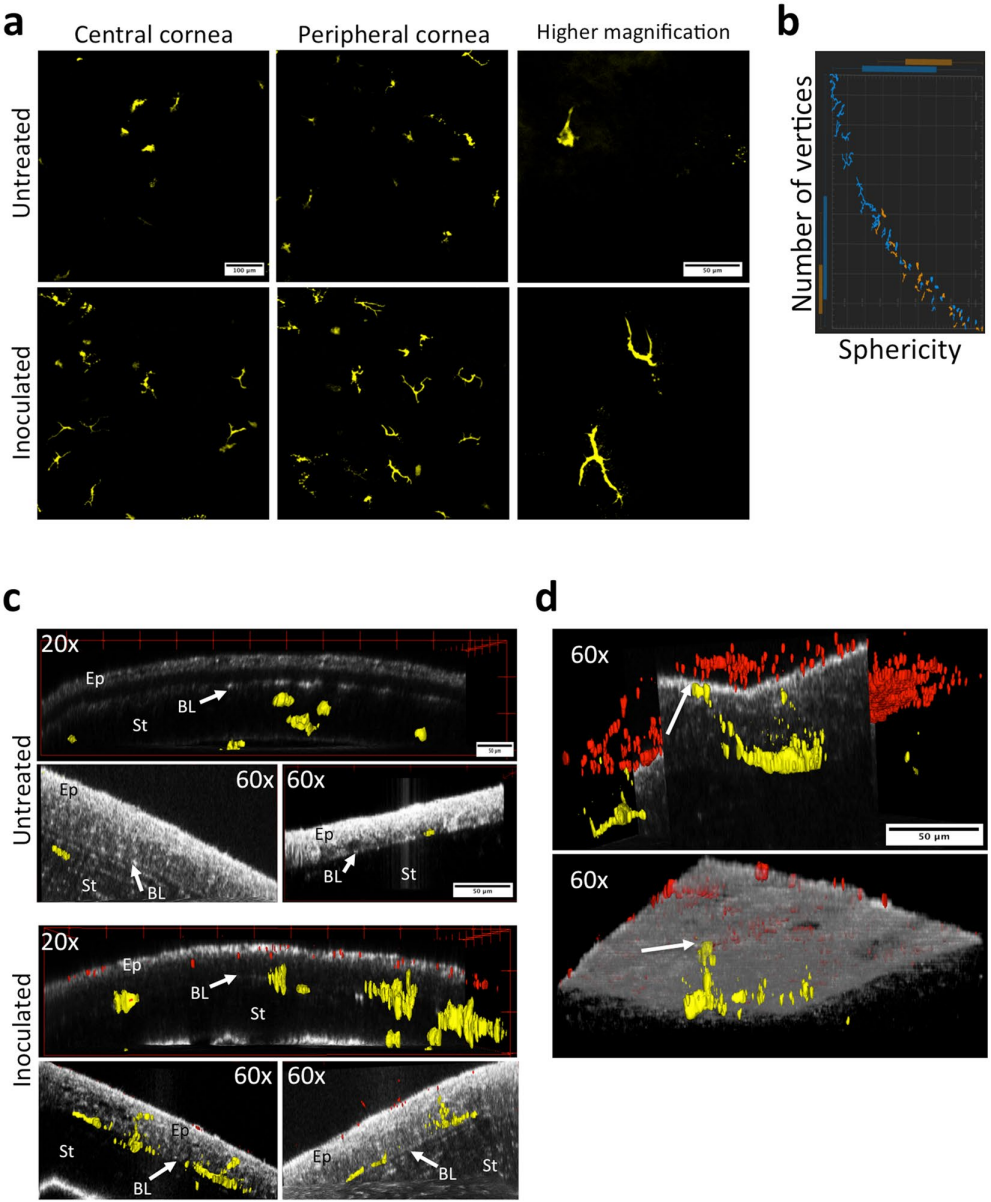
Morphological changes in murine CD11c+ cells upon *P. aeruginosa* challenge *in vivo*. (**a**) Maximum intensity projection of YFP signal (yellow, CD11c+ cells) in uninjured mouse corneas 4 h after challenge with PAO1 *in vivo*. CD11c+ from corneas exposed to bacteria show long dendritic processes contrasting with a more globular shape in untreated controls. Images from the central and peripheral cornea (20x objective), and higher magnification (60x objective). (**b**) Graphical representation of the distribution of morphological differences between CD11c+ cells in control (orange) versus bacteria-exposed (blue) corneas using Imaris software. (**c**) CD11c+ cells approached the apical surface of the corneal epithelium (EP) inoculated with PAO1 in contrast with untreated controls where CD11c+ cells localized mostly below the basal lamina (BL) in the stroma (ST). YFP-CD11c+ cells (yellow) and dTomato-PAO1 (red) are shown using 3-dimensional rendering. Corneas are shown as orthogonal optical sections to allow proper CD11c+ cell visualization. (**d**) When the mouse cornea is superficially-injured (tissue paper blotted), CD11c+ cells can extend processes to reach the epithelial surface. Two examples are shown of CD11c+ cells sending processes that reach the epithelial surface in close proximity with *P. aeruginosa* adhering to the epithelium (arrows) (60x objective). YFP-CD11c+ cells (yellow) and dTomato-PAO1 (red) are shown using 3-dimensional rendering. The epithelial apical surface is shown as an orthogonal optical section (top image) and volume (bottom image) to allow proper localization of CD11c+ cells and bacteria.

Given the observed contribution of bone marrow-derived cells to corneal epithelial barrier function against bacterial adhesion, the established role of dendritic cells (CD11c+) in sampling other mucosal surfaces^16,17^, and the known presence of dendritic cells in a healthy cornea^19^, we utilized CD11c-YFP transgenic mice to explore if CD11c+ cells responded to *P. aeruginosa* when the cornea is healthy. *In vivo* exposure of healthy corneas to *P. aeruginosa* for only 4 h caused CD11c+ cells in central and peripheral regions of the cornea to produce long dendritic processes contrasting with the more globular shape observed in control uninoculated eyes (Fig. 4a). Consistent with these observations, CD11c+ cells within inoculated corneas showed lower sphericity and more vertices compared to controls (Fig. 4b, Supplemental Fig. S1). In 3D reconstructions of z-stacks, we observed CD11c+ cells localizing closer to the epithelial surface (Fig. 4c, 20x panels) with dendritic processes extending into the epithelium (Fig. 4c, 60x panels) of the inoculated corneas, in contrast with the sub-basal and stromal localization of CD11c+ cells in uninoculated controls. When corneas were blotted *in vivo* before bacterial inoculation, CD11c+ cell processes actually reached the epithelial surface in close proximity to colonizing bacteria (Fig. 4d, Supplemental Movie S3). Importantly, when bacteria were added to eyes *ex vivo* rather than *in vivo*, there was no observable change in CD11c+ cell morphology or localization (data not shown). Together these data show that corneal CD11c+ cells sense, respond, and migrate towards, bacteria with and without injury, and that *in vivo* factors beyond the eye contribute to the mechanisms involved.

### CD11c+ cell depletion increases *P. aeruginosa* adhesion to the cornea after superficial injury

**Figure 5:**
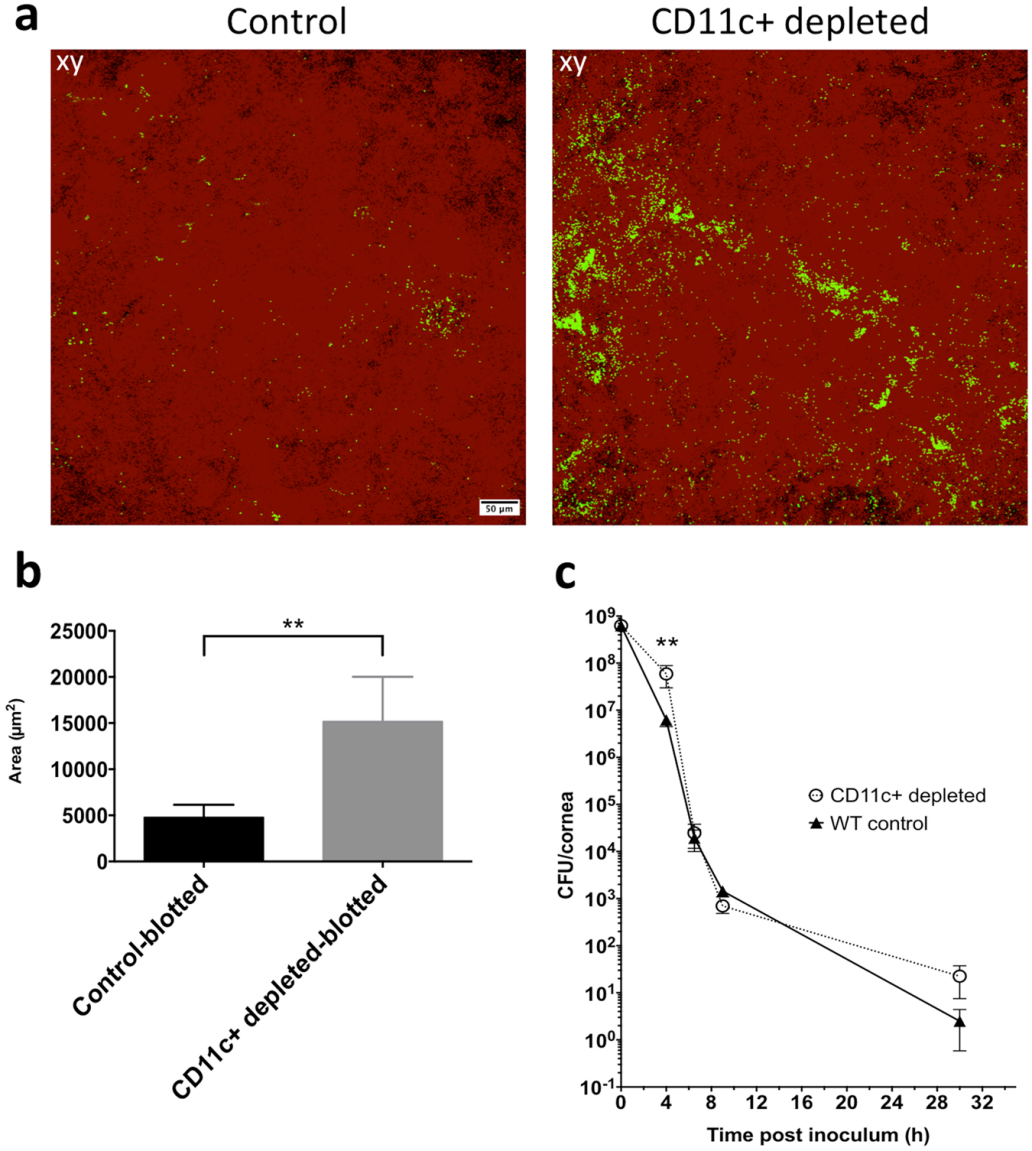
CD11c+ cells help prevent bacterial adhesion to the cornea after superficial-injury *in vivo*. (**a**) Maximum intensity projection of tissue paper blotted corneas of CD11c+ cell-depleted mice showing increased *P. aeruginosa* adhesion compared to controls (injected with saline 0.9% instead of diphtheria toxin) after 4 h *in vivo*. (**b**) Quantification of adherent bacteria from 4 or more fields per eye, and three biological replicates under experimental conditions in A, showing > 3-fold increase in bacterial adhesion to CD11c+ cell-depleted corneas versus controls (**p < 0.01, Mann-Whitney test). Corneal epithelium is shown in red (reflection), and bacteria are green (GFP), 20x objective. (**c**) *In vivo* bacterial clearance from CD11c+ cell-depleted corneas. Viable counts (CFU/cornea) of ocular eye washes from CD11c+ cell-depleted mice (open circles) compared to controls (full triangles). **p < 0.01, two-way ANOVA with Sidak’s multiple comparison test.

Since CD11c+ cells in healthy corneas appeared to be activated by *P. aeruginosa* challenge, we explored if they played a role in barrier function. Diphtheria Toxin Receptor (DTR) transgenic mice were used to transiently deplete CD11c+ cells (see Methods) 20 h prior to bacterial challenge *in vivo* to determine if bacterial adhesion or penetration were affected. We first studied uninjured corneas, and found that CD11c+ cell-depleted mice showed similar *P. aeruginosa* adhesion to respective controls (Supplemental Fig. 2a). However, after blotting injury, the corneas of CD11c+ cell-depleted mice showed a significant increase (>3-fold) in *P. aeruginosa* adhesion compared to controls (Fig. 5a and b). Bacterial penetration beyond the surface was not observed in either group. To examine if CD11c+ cell depletion also affected the capacity of the ocular surface to clear bacteria, eyewashes were collected up to 30 h post-inoculation *in vivo*, and viable counts were performed. CD11c+ cell-depleted mice showed a significant reduction in the ability to clear bacteria after 4 h, but no significant differences were observed at later time points (Fig. 5c) with complete clearance after 30 h (data not shown). When these experiments were performed *ex vivo* rather than *in vivo*, no differences in adhesion were observed between CD11c+ cell-depleted corneas and controls (with or without blotting) (Supplemental Fig. 2b), and bacteria did not penetrate the epithelium (data not shown). These *ex vivo* findings were expected based on the lack of visible CD11c+ cell responses to the bacterial inoculum *ex vivo* previously discussed.

### *In vivo* transcriptional changes in the corneas of wild-type C57BL/6 versus IL-1R (−/−) mice before and after *P. aeruginosa* challenge

**Figure 6:**
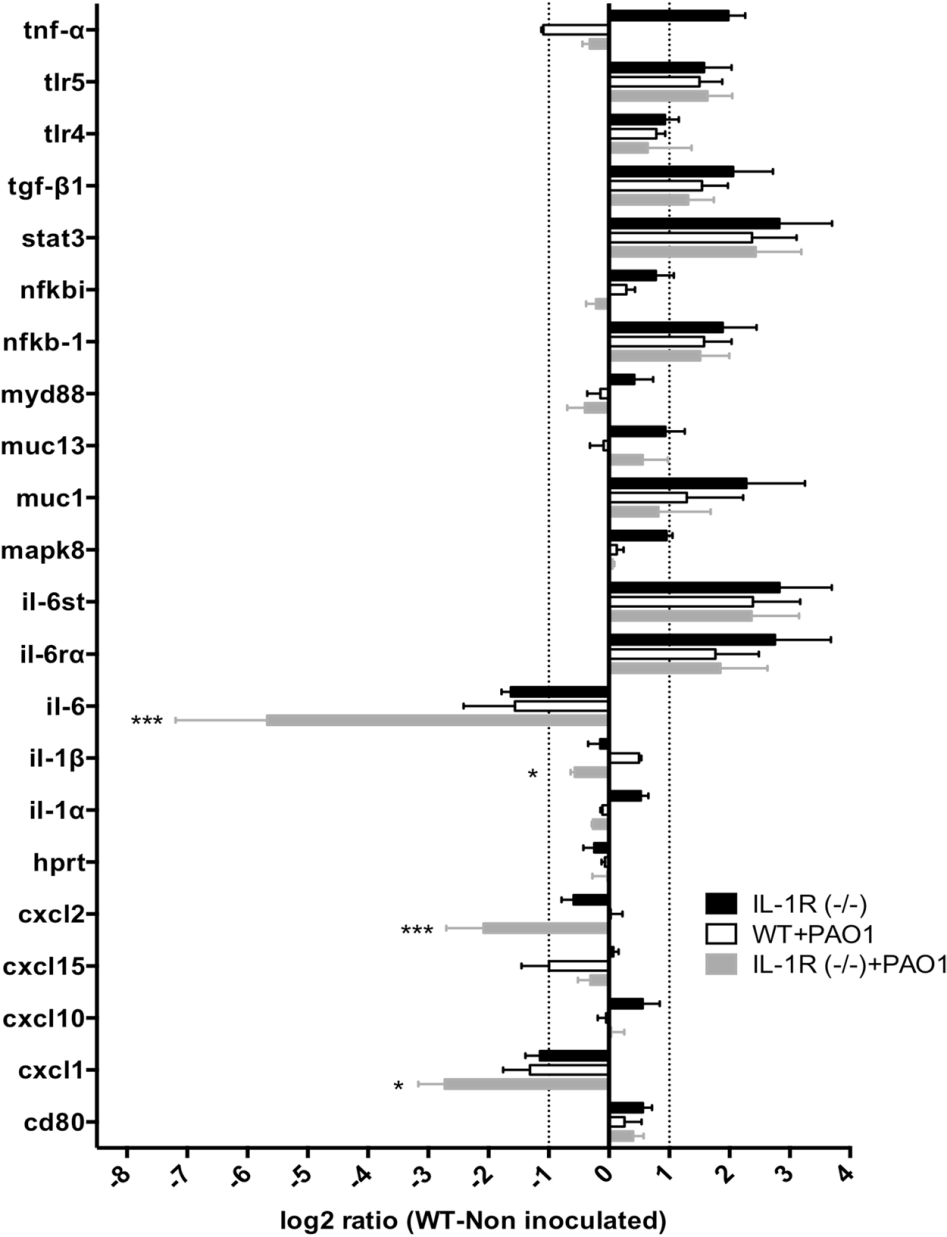
Transcriptional analysis of host cell gene expression in WT and IL-1R (−/−) murine corneas *in vivo* with or without 4 h challenge with *P. aeruginosa* strain PAO1. qRT-PCR results are shown as a log_2_ ratio relative to uninoculated WT corneas (normalized to zero). Transcription of selected gene targets in uninoculated IL-1R (−/−) corneas (black bars) was compared to PAO1 challenged WT (white bars) or IL-1R (−/−) (grey bars). Dotted lines indicate 2-fold difference in expression (log_2_ ratio ±1) compared to uninoculated WT. *p < 0.05, ***p < 0.001, inoculated IL-1R (−/−) (grey bars) versus inoculated WT (white bars), two-way ANOVA with Sidak’s multiple comparison test.

Given the prominent role of IL-1R in preventing bacterial adhesion in healthy corneas and penetration of the blotted corneal epithelium *in situ*, a transcription array was used to compare expression of genes related to bacterial recognition and response in wild-type and IL-1R (−/−) healthy corneas before and after *P. aeruginosa* challenge for 4 h *in vivo* (Fig. 6). Prior to inoculation, expression of several genes was altered 2-fold or more (log_2_ ≥ + /−1) in IL-1R (−/−) corneas relative to wild-type. These included; increased expression of TNFα, TLR5, TGFβ-1, Stat-3, NFκB-1, Muc1, the IL-6 signal transducer, and IL-6 receptor α, and decreased expression of IL-6 and CXCL1 (Fig. 6, black bars). Except for TNFα, each of those genes in uninoculated IL-1R (−/−) corneas showed a similar pattern of expression to wild-type inoculated with *P. aeruginosa* (Fig. 6, black bars versus white bars). Comparison of gene expression after *P. aeruginosa* challenge revealed significantly lower expression of IL-6, CXCL1, CXCL2 and IL-1β in IL-1R (−/−) versus wild-type mice (Fig. 6, grey bars versus white bars).

### Corneal gene expression after CD11c+ cell depletion and bacterial challenge *in vivo*

**Figure 7:**
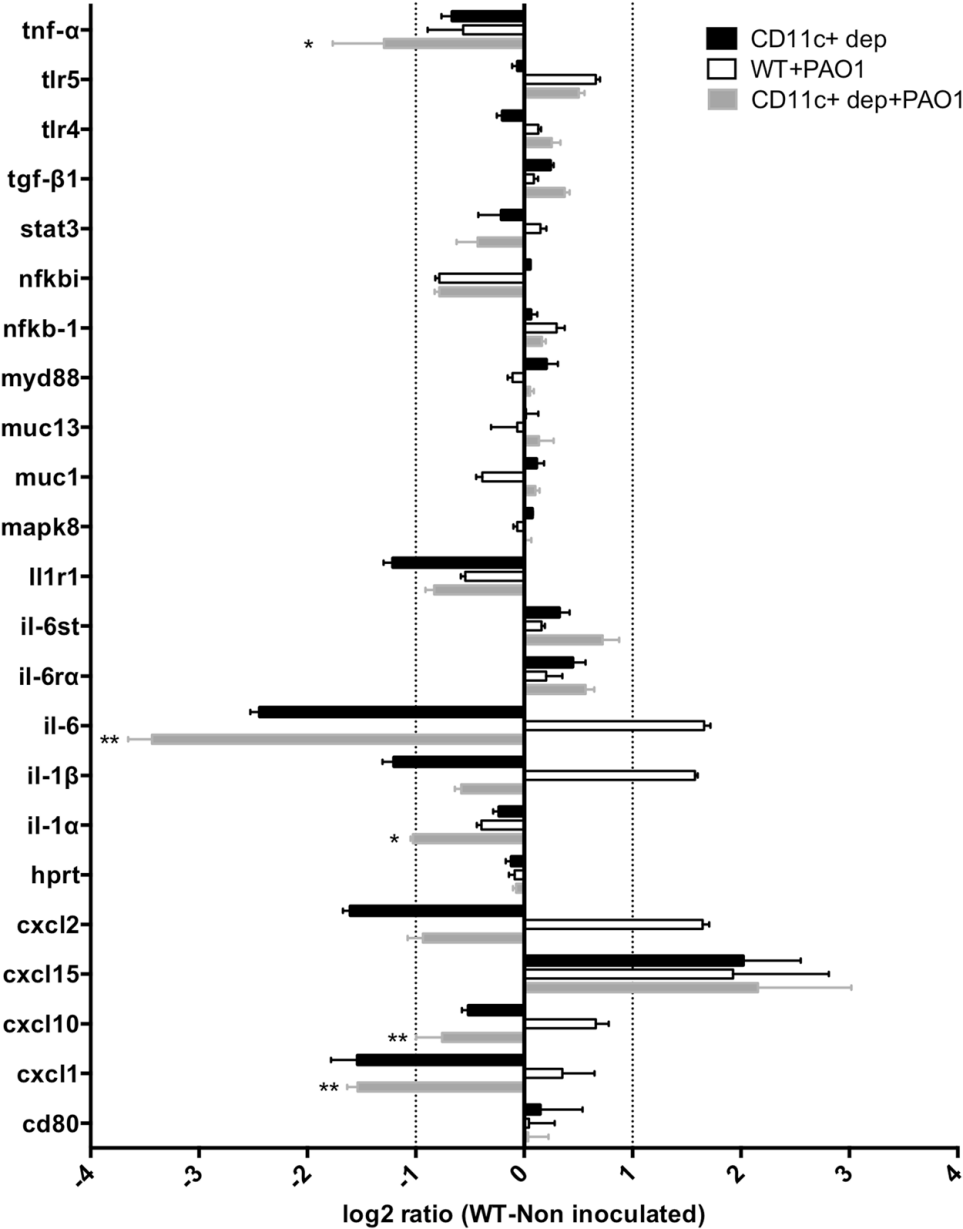
Transcriptional analysis of host gene expression in tissue paper blotted (superficially- injured), CD11c+ cell-depleted, murine corneas *in vivo* with or without a 4 h challenge with *P. aeruginosa* strain PAO1. qRT-PCR results are shown as a log_2_ ratio relative to uninoculated, blotted control corneas (normalized to zero). Transcription analysis of selected genes in uninoculated, blotted, CD11c+ cell-depleted corneas (black bars) was compared to inoculated, blotted, CD11c+ cell-depleted corneas (grey bars) and inoculated, blotted controls (white bars). Dotted lines indicate a 2-fold difference in expression (log_2_ ratio ±1) compared to uninoculated, blotted controls. *p < 0.05, **p < 0.01, inoculated, blotted, CD11c+ cell-depleted corneas (grey bars) versus inoculated, blotted controls (white bars), two-way ANOVA with Sidak’s multiple comparison test.

To better understand how CD11c+ cell depletion increased *P. aeruginosa* adhesion to tissue paper blotted corneas *in vivo*, the transcriptional profile of genes related to bacterial recognition and response in these corneas was examined, and compared to blotted control corneas after bacterial exposure, and blotted CD11c+ cell-depleted or control corneas without bacterial exposure. Blotted, CD11c+ cell-depleted corneas without bacterial exposure showed a 2-fold or more decrease in expression of genes encoding IL-1R, IL-6, IL-1β, CXCL1 and CXCL2 compared to blotted, uninoculated controls, and an increase in CXCL15 gene expression (Fig. 7, black bars). Comparison of transcriptional profiles of inoculated corneas of blotted, CD11c+ cell-depleted mice (Fig. 7, grey bars) with inoculated, blotted controls (Fig. 7, white bars) showed opposite patterns of change (i.e. increased expression for inoculated controls; decreased expression for inoculated CD11c + cell-depleted corneas) for IL-6, IL-1β, CXCL1, CXCL2, and CXCL10. Significant decreases in gene expression were also observed for TNFα and IL-1α in inoculated, blotted, CD11c+ cell-depleted corneas compared to inoculated, blotted controls (Fig. 7). These data suggest a major role for resident CD11c+ cells in maintaining a steady-state level of cytokines and chemokines during health, and inducing their production when the corneal epithelium is in close contact with *P. aeruginosa*.

## Discussion

It is well established that MyD88-dependent receptors participate in inflammatory and immune responses of the murine cornea to *P. aeruginosa* infection. Here, we demonstrated an additional and novel function for MyD88-dependent receptors in maintenance of corneal epithelial barrier function to *P. aeruginosa* during health. As summarized in Table 1, the data show that TLR4 and IL-1R function to prevent *P. aeruginosa* adhesion to uninjured murine corneas, and that IL-1R expressed by both bone marrow-derived and non-bone marrow-derived cells participated in that defense. The data also show that IL-1R and TLR5 prevent *P. aeruginosa* penetration through the murine corneal epithelium when it was superficially-injured, and that IL-1R expressed only in non-bone marrow-derived cells was sufficient, as shown by chimera studies. However, we also showed that resident CD11c+ cells *in vivo* sensed and responded to bacteria on the healthy corneal surface, and also after superficial epithelial injury (tissue paper blotting). CD11c+ cells spatially interacted with bacteria, reducing *P. aeruginosa* adhesion to the cornea as shown using CD11c+ cell-depleted mice. Related to this, transcriptional analysis revealed significant reductions in IL-6 and CXCL1 gene expression in IL-1R (−/−) mice relative to wild-type, with enhanced differences upon bacterial challenge. Similarly, CD11c+ cell depletion also led to a loss of corneal epithelial gene responses to *P. aeruginosa* challenge after blotting, including decreased IL-6, IL-1β, CXCL1, CXCL2 and CXCL10, which might relate to increased bacterial adhesion noted with CD11c+ cell depletion.

**Table 1:** Summary of *P. aeruginosa* PAO1 adhesion to, and penetration of, murine corneal epithelia *ex vivo* and *in vivo*

C57BL/6 Murine Genotype	Healthy cornea		Superficially-injured cornea	
	Bacterial adhesion	Bacterial penetration	Bacterial adhesion	Bacterial penetration
*Ex vivo*				
TLR4 (−/−)	+	None	++	None
TLR5 (−/−)	−	None	n/d	Yes
IL-1R (−/−)	++	None	n/d	Yes
WT/IL-1R (−/−)bm	+	None	n/d	None
IL-1R (−/−)/WTbm	+	None	n/d	Yes
CD11c+ cell-depleted	−	None	−	None
*In vivo*				
CD11c+ cell-depleted	−	None	++	None

++ or +, significantly greater than WT control (p < 0.01 or p < 0.05 respectively). −, no significant difference versus WT control. n/d, Not done.

TLRs and IL-1R have previously been shown to be involved in inflammatory responses^11–14^. For example in a corneal abrasion infection (i.e. disease) model, TLR4 and TLR5 were fundamental for host responses to *P. aeruginosa* LPS and flagellin respectively *in vivo*; responses attributed predominantly to stromal macrophages^21^. Similarly for corneal epithelial cells grown *in vitro*, TLR5-mediated inflammatory responses to *P. aeruginosa* flagellin^22^, and either *P. aeruginosa* LPS (a ligand for TLR4) or IL-1β (a ligand for the IL-1R) increased the expression of human β-defensin 2, an important antimicrobial peptide^23,24^. However, the role of these receptors in the maintenance of epithelial barrier function against *P. aeruginosa* in a healthy cornea *in situ* has not been explored.

The mechanisms by which TLR4, TLR5 and IL-1R prevent bacterial adhesion and/or epithelial penetration in our results *ex vivo* could involve the expression of antimicrobial peptides by epithelial cells. Several TLRs, IL-1β, and MyD88 play roles in expression of antimicrobial peptides by corneal epithelial cells^24–26^. Our previous work has shown that both mBD3 (murine equivalent of hBD2) and novel keratin-derived antimicrobial peptides contribute to defending the corneal epithelium against *P. aeruginosa* adhesion *in vivo*, and hBD2 protects against epithelial penetration *in vitro* ^3,4^. Other candidate factors modulated by TLRs and IL-1R include the highly glycosylated mucins. Indeed, we have previously shown that mucins can protect against *P. aeruginosa* adhesion to the corneal epithelium^5,27^, and more recently that constitutive corneal surface glycosylation requires IL-1R^28^. Epithelial barrier function against *P. aeruginosa* can also be influenced by the integrity of tight junctions or cell polarity. In this respect, TLR2 has been shown to modulate tight junction integrity in the intestinal tract and dermis^29–32^, and we have previously shown that polarized cells are less susceptible to *P. aeruginosa* invasion^33,34^.

Activation of TLR5 and IL-1R by their ligands (flagellin and IL-1 respectively) also induces the production of pro-inflammatory cytokines and chemokines in corneal epithelial cells^22,35^. Inflammatory mediators released prior to infection, could directly influence corneal epithelial defense against *P. aeruginosa* adhesion and penetration, e.g. *via* antimicrobial peptide expression, or indirectly by recruiting myeloid-derived cells^15^ discussed further below. In this respect, the transcriptional profile of healthy IL-1R (−/−) corneas even when not inoculated revealed an overall signature similar to bacteria-inoculated WT corneas for several genes related to inflammatory and immune responses. These included an upregulation of genes encoding TLR5, TGFβ-1, Stat-3, NFκB-1, IL-6 signal transducer and IL-6 receptor α, suggesting a more “pro-inflammatory state” even prior to bacterial challenge. Upon *P. aeruginosa* challenge, those same genes did not respond, suggesting an impairment in sensing and responding to microbial components at the epithelial surface. Interestingly, bacteria inoculated IL-1R (−/−) corneas showed significantly lower expression of genes encoding IL-6, CXCL1 and CXCL2 compared to uninoculated IL-1R (−/−). The latter was similar to inoculated WT with both showing lower expression of these mediators than uninoculated WT.

Similarly, transcriptional analysis of blotted CD11c+ cell-depleted corneas without bacteria revealed significantly lower expression of IL-1R, IL-6, IL-1β, CXCL1, CXCL2 consistent with the loss of CD11c+ cells. The observation that bacterial challenge of superficially-injured (blotted) corneas *in vivo* induced opposing patterns of expression for IL-6, IL-1β, CXCL1, CXCL2, and CXCL10 in WT (increased expression) versus CD11c+ cell-depleted (decreased expression) is consistent with our data showing that CD11c+ cell depletion reduces defense against *P. aeruginosa* adhesion. These cytokines and chemokines are known to be important for the activation of inflammatory and immune responses during an infection, including myeloid cell recruitment in the cornea and other tissues. Interestingly, IL-6 and CXCL1 can be secreted by intestinal stromal cells or macrophages in an IL-1R-dependent manner to recruit neutrophils^36,37^, and neutrophils were found in the murine cornea after challenge with *P. aeruginosa* Outer Membrane Vesicles (OMVs) *in vivo* even without infection^38^. CXCL10 (IP-10) is a chemo-attractant for CXCR3+ cells including dendritic cells, T lymphocytes, natural killer cells, and other immune cells^39^, and also secreted in a MyD88-dependent manner during Chlamydial infection, albeit independently of TLR2 and TLR4^40^. IL-6 is upregulated on corneal epithelial and myeloid cells during *P. aeruginosa* infection^41^, and the role of IL-1β in defense against *P. aeruginosa* corneal infections has been demonstrated^42,43^, including a recently demonstrated role in ocular and gut microbiota-driven protection of the cornea against *P. aeruginosa* ^44^.

A *caveat* to the present study is that only transcriptional changes were shown using our *in vivo* models. It remains to be determined if changes in host cell gene expression translate into differences in protein expression and/or secretion. Nevertheless, our data suggest an important new role for IL-1R and CD11c+ cells in modulating mediators of constitutive innate defense of healthy and superficially-injured corneas, and their responses to *P. aeruginosa* in the absence of active infection.

Studies using disease models that bypass the epithelial barrier have shown a primary role for myeloid cells (mostly dendritic cells and macrophages)^13,45^, which participate in responding to *P. aeruginosa* keratitis once disease is underway (after bacteria enter the stroma)^12,21^. It remains to be determined if one or more of these myeloid cell types contribute to the role of CD11c+ cells in sensing and responding to bacteria when the cornea is healthy (i.e. when barrier function is operational).

The important role of CD11c+ cells in constitutive defense, suggested by alterations in transcriptional profile after depletion, was further supported by morphological changes, and their recruitment to the healthy corneal epithelium after bacterial exposure, demonstrating an active role in surveying the ocular surface. However, it is not yet known whether CD11c+ cells directly sense bacterial components on the epithelial surface, or if they receive signals from the epithelium, or from other sites on the ocular surface. Intriguingly, CD11c+ cells were responsive to the presence of bacteria only if inoculation was performed *in vivo*, suggesting involvement of other factors or systems that are compromised *ex vivo*, e.g. lymphatic vessels, conjunctival drainage, tear fluid or corneal nerves. Interestingly, it was recently shown that dendritic cells (CD11c+) closely interact with nerves in the murine cornea^46^, which could help facilitate the CD11c+ cell responses observed. CD11c+ cell-depletion experiments also showed that resident CD11c+ cells were involved in defending blotted corneas against *P. aeruginosa* adhesion *in vivo*. The close proximity of inoculated bacteria with CD11c+ cell processes that extended to the epithelial surface in blotted corneas suggested that direct interaction mediated the reduction in adhesion. It is not yet clear, however, if the CD11c+ cell contribution to reduced adhesion involves bacterial killing, e.g. by phagocytosis or antimicrobial peptide activity, or another means of preventing bacteria-epithelial cell interactions. The lack of difference in bacterial clearance between CD11c+ cell-depleted and control eyes at time points longer than 4 h (i.e. after mice awake from anesthesia) might reflect the transient nature of CD11c+ cell depletion^47^, along with the presence of additional clearance mechanisms (e.g. tear fluid) that may compensate for the absence of CD11c+ cells.

It is important to emphasize that TLR5 (−/−) or CD11c+ cell-depleted uninjured corneas did not show an increase in bacterial adhesion. In addition, despite the increase of adherent bacteria in blotted CD11c+ cell-depleted corneas, or in both blotted and non-blotted TLR4 (−/−) corneas, we did not observe *P. aeruginosa* penetration of the epithelium. Finally, while IL-1R expression from both bone marrow-derived, and non-bone marrow-derived, cells contributed to defense against adhesion, only non-bone marrow-derived IL-1R was involved in preventing bacterial penetration. Together these findings suggest a clear distinction between the events (and consequent defenses) that underline initial bacterial adhesion, and those associated with subsequent bacterial penetration into the corneal epithelium. IL-1R expression is involved in both, but with different contributions from different cell types.

In conclusion, this study suggests that the healthy cornea is in constant surveillance of the environment and senses (via CD11c+ cells) potential threats at its surface to prevent bacterial adhesion (TLR4, IL-1R, CD11c+ cells) and block epithelial penetration (TLR5, and non-bone marrow-derived cell IL-1R). Thus, we show novel roles for these receptors and cells in sensing and preventing bacterial interaction with the cornea to prevent infection. A better understanding of how mucosal surfaces maintain their health in environments that are continuously exposed to potential pathogens is a key step in deciphering the mechanisms by which normally resistant tissues become susceptible to infection, and how pathogens circumvent those defenses.

## Methods

### Bacteria

*Pseudomonas aeruginosa* strain PAO1 expressing enhanced GFP (PAO1-GFP) or d-Tomato was used^48,49^. Bacteria were grown on tryptic soy agar (TSA) plates supplemented with carbenicillin 300 µg/mL at 37 °C for 16 h. Inocula were prepared by suspending bacteria in Dulbecco’s Modified Eagle’s Medium (DMEM) (Lonsa, Walkersville, MD) to a concentration of ~10^11^ CFU/mL.

### *Ex vivo* murine model for bacterial adhesion and penetration of the corneal epithelium

All animals were treated according to the ARVO statement for use of animals in Ophthalmic Research. All procedures were approved by the Animal Care and Use Committee, University of California, Berkeley, an AAALAC accredited institution. Gene knockout C57BL/6 mice in TLR-2, TLR-4, TLR-5, TLR-7, and TLR-9 were provided by Dr. Greg Barton (University of California, Berkeley, CA). IL-1R (−/−) (#003245), CD11c-YFP (#008829), DTR (#004509) and Ubi-GFP (#004353) and age and sex matched C57BL/6 wild-type mice (#000664) were obtained from the Jackson Laboratory (Bar Harbor, ME). The *ex vivo* model was used as described previously^9^. Mice were subject to intraperitoneal injection with a lethal dose of anesthetic cocktail containing ketamine (240 mg/Kg) and xylazine (30 mg/Kg) followed by cervical dislocation. Whole eyes were enucleated and washed in PBS to exclude tear fluid before tissue paper blotting with a Kimwipe^TM^. Alternatively, eyes were washed in PBS and not blotted (uninjured). Eyes were inoculated with 200 µl of *P. aeruginosa* suspension (~10^11^ CFU/mL in DMEM) and incubated for 6 h at 35 °C before imaging.

### Generation of bone marrow chimeras

Bone marrow chimera mice were generated as described in^50^ with minor modifications. Briefly, 6 weeks old donor mice were anesthetized using 5% isofluorane and sacrificed by cervical dislocation. The mouse skin was extensively disinfected using 96% ethanol, using a sterile scalpel femurs and tibias were separated from the muscles, washed in 96% ethanol for 60 seconds and rinsed in ice-cold PBS. Bone marrow cells were extracted by flushing PBS into the bones using a sterile 25-G needle after cutting the distal and proximal epiphyses. Erythrocytes were removed from the extracted bone marrow using RBC lysis buffer (Sigma), and the suspension was then filtered through a 40 µm cell strainer (Falcon). Live cells were enumerated with Trypan blue, and resuspended in PBS at a concentration of 1 × 10^8^/mL. Recipient mice (6–7 weeks old) were subjected to 1200 rads lethal dose (600+ 600 with a 4 h interval) whole body irradiation using a Precision X-Ray machine (X-RAD 320) and subsequently injected through the tail vein with 100 µl of bone marrow preparation (~10^7^ cells). Mice were given antibiotic-supplemented water (trimethoprim/sulfamethoxazole) for 3 weeks after irradiation to allow time for bone marrow reconstitution. After one week without antibiotics, mice were used in the *ex vivo* adhesion/penetration model described earlier. As a control, bone marrow from Ubi-GFP transgenic mice was injected into irradiated WT recipient mice to confirm reconstitution with the donor genotype (green bone marrow derived cells present in the cornea stroma, data not shown).

### *In vivo* bacterial adhesion model and CD11c+ cell depletion

CD11c+ cell morphology and interactions with bacteria were examined *in vivo* using CD11c-YFP C57BL/6 mice (yellow fluorescent CD11c+ cells) and PAO1-dTomato (red fluorescent *P. aeruginosa*). CD11c+ cell depletion in murine corneas was achieved using CD11c-DTR/GFP C57BL/6 mice and intraperitoneal injection with diphtheria toxin (4 ng/g of body weight, Sigma) 20 h prior to bacterial challenge. Control mice were injected with the same volume of sterile saline (0.9% wt./vol.). Mice were anesthetized using a cocktail of ketamine (80 mg/Kg) and dexemedetomide (0.5 mg/Kg). One cornea of each anesthetized mouse was rinsed with PBS to wash away tear fluid, blotted with tissue paper to enable bacterial adhesion, and inoculated with 5 μl of *P. aeruginosa* (~10^11^ cfu/mL in DMEM). After 4 h, animals were euthanized, eyes enucleated, rinsed with PBS, and corneas either isolated for RNA extraction (Trizol^TM^) or imaged using confocal microscopy. To determine bacterial adhesion to the murine ocular surface over time, inoculated eyes were washed (with the mice under isofluorane anesthesia) at indicated time points using 10 µl of PBS gently pipetted onto one extremity of the eye, and rapidly collected on the other extremity using a capillary tube. Eyewashes were then diluted and plated on TSA agar for CFU counts. After CD11c+ cell depletion, eyes were routinely imaged for absence of a GFP signal, confirming depletion of GFP-CD11c+ cells.

### Confocal microscopy

After treatments, mouse eyeballs were glued to a Petri dish with the cornea facing upwards, and submerged in clear DMEM (without phenol red). Confocal imaging used a 60x/1.00 NA or a 20x/0.56 NA water-dipping objective and an upright Olympus Fluoview FV1000 Confocal Microscope. Eyes where imaged using reflection 635 nm (corneal cells), 559 nm (PAO1-dTomato), 515 nm (YFP-CD11c+ cells), and 488 nm (PAO1-GFP) lasers. Z stacks (1.0 µm steps) were collected from 4 or more random fields per sample. Maximum intensity projection (reducing a 3D image into 2D by projecting the maximum intensity of each pixel in a specific channel to the z plane [xy]) was used to visualize colonizing bacteria, and optical orthoslicing was used to visualize traversing bacteria (2 µm thick optical section, yz). 3-D image reconstruction and cell morphology analysis was performed by Image-J. Bacterial adherence was defined as the total area (µm^2^) occupied by the GFP or dTomato signal on the corneal surface for each field. Each experiment involved four different samples and was performed at least three times.

### RT-qPCR

After *in vivo* treatments, corneas were carefully dissected to remove all limbal tissue, and RNA rapidly extracted using Trizol prior to cDNA synthesis and RT-qPCR. Corneas from freshly enucleated eyes (three or more per condition) were also carefully dissected using a sterile scalpel and rapidly placed in ice-cold Trizol (Thermo Fisher Scientific). Corneas were disrupted using a combination of manual grinding with a mortar and pestle, and a hand-held tissue homogenizer (Kinematica Polytron, Thermo Fisher Scientific). RNA was extracted from the homogenate in Trizol according to manufacturer’s instructions, and cDNA synthesis performed using iScript (Bio-Rad) and RT-qPCR using Faststart Sybergreen (Roche) running on a Light Cycler 96 real-time PCR machine (Roche).

Targets in the gene panel were selected according to known involvement in antibacterial response, TLR signaling and inflammation. RT-PCR primers were selected from the mouse qPCR primers database accessible through the UCSC genome browser, and from primerBank (https://pga.mgh.harvard.edu/primerbank/index.html) when available, or designed using NCBI Primer-BLAST (https://www.ncbi.nlm.nih.gov/tools/primer-blast/). Primers were designed to be separated by at least one intron to assure selective amplification of cDNA, and tested for efficiency (greater than or equal to 1.90), and specificity under conditions used. Gene targets and primer sequences are listed in Table 2. To control for the effects of diphtheria toxin alone, RT-qPCR analysis was performed on corneas from WT mice injected with the toxin (with or without bacterial exposure), and compared to controls that did not receive toxin. There were no significant differences in the expression of selected genes between toxin injected mice and controls (data not shown).

**Table 2:** Primers used in this study.

Gene name	Source	Forward (5′-3′)	Reverse (5′-3′)
muc1	NCBI	GCA TTC GGG CTC CTT TCT TC	CCT CAC TTG GAA GGG CAA GA
muc13	NCBI	CTT CTG CAA TCG AAA CTG CAA	ATG TCC TGG CAT TTA CTG CTG
cd80	PB	GCT GTG TCG TTC AAA AGA AGG A	TGG GAA ATT GTC GTA TTG ATG CC
cxcl1	PB	CTG GGA TTC ACC TCA AGA ACA TC	CAG GGT CAA GGC AAG CCT C
cxcl10	PB	CCA AGT GCT GCC GTC ATT TTC	GGC TCG CAG GGA TGA TTT CAA
cxcl15	PB	CAA GGC TGG TCC ATG CTC C	TGC TAT CAC TTC CTT TCT GTT GC
cxcl2	NCBI	AGT GAA CTG CGC TGT CAA TG	TCA GTT AGC CTT GCC TTT GTT C
gapdh	NCBI	TGC GAC TTC AAC AGC AAC TC	GCC TCT CTT GCT CAG TGT CC
hprt	NCBI	TCA GTC AAC GGG GGA CAT AAA	GGG GCT GTA CTG CTT AAC CAG
il-1α	NCBI	AGT CGG CAA AGA AAT CAA GAT G	CCT TGA AGG TGA AGT TGG ACA
il-6	NCBI	CCT CTC TGC AAG AGA CTT CCA TC	CCA TTG CAC AAC TCT TTT CTC A
il-1β	PB	CAA CCA ACA AGT GAT ATT CTC CAT G	GAT CCA CAC TCT CCA GCT GCA
il-1r1	PB	GTG CTA CTG GGG CTC ATT TGT	GGA GTA AGA GGA CAC TTG CGA AT
il-6rα	UCSC	TGC TCC CTG AAT GAT CAC CT	TCA CAG ATG GCG TTG ACA AG
il-6st	UCSC	GTG AAT CGG ACC CAC TTG AG	GGC GAA TAC GGG AGT TAC TGT
mapk8	UCSC	AAT CAG ACC CAT GCT AAG CG	TGA AAA CAT TCA AAA GGC CAA
nfkb1	UCSC	GAC CCA AGG ACA TGG TGG T	ATC CGT GCT TCC AGT GTT TC
nfkbi	NCBI	TCG CTC TTG TTG AAA TGT GG	TCA TAG GGC AGC TCA TCC TC
stat3	PB	AGC TGG ACA CAC GCT ACC T	AGG AAT CGG CTA TAT TGC TGG T
tgf-β 1	UCSC	GCA ACA TGT GGA ACT CTA CCA GA	GAC GTC AAA AGA CAG CCA CTC A
tlr4	NCBI	CAG CAA AGT CCC TGA TGA CA	AGA GGT GGT GTA AGC CAT GC
tlr5	PB	GCA GGA TCA TGG CAT GTC AAC	ATC TGG GTG AGG TTA CAG CCT
tnf-α	NCBI	AGG GAT GAG AAG TTC CCA AAT G	CAC TTG GTG GTT TGC TAC GAC

NCBI: NCBI Primer-BLAST, PB: primerBank, UCSC: mouse qPCR primers database.

### Statistical analysis

Data were expressed as medians with interquartile ranges except for fold-difference changes in adhesion expressed as mean+/− standard deviation. Statistical significance of differences between three or more groups was determined using the Kruskal-Wallis test with Dunn’s multiple comparison test for post-hoc analysis, or by two-way ANOVA with Sidak’s multiple comparison test. The Mann-Whitney U test or Student’s t-Test were used to compare two groups. P values of less than 0.05 were considered significant.

### Data availability

All data generated or analyzed in this study are included in this published article (and its Supplementary Information files).

## Electronic supplementary material

Supplementary Materials

Murine cornea CD11c+ cells interacting with P. aeruginosa in vivo
