# Supplementary material for: Contributions of MyD88-dependent receptors and CD11c-positive cells to corneal epithelial barrier function against Pseudomonas aeruginosa
Source: Sci Rep. 2017 Oct 23;7:13829. doi: 10.1038/s41598-017-14243-w (PMC5653778; doi:10.1038/s41598-017-14243-w)
Supplement: Supplementary file 1 — Supplementary Materials [file 41598_2017_14243_MOESM1_ESM.pdf]

Contributions of MyD88-dependent receptors and CD11c-positive cells to corneal epithelial barrier function against *Pseudomonas aeruginosa*

Matteo M.E. Metruccio, Connie Tam, David J. Evans, Anna L. Xie, Michael E. Stern, and Suzanne M.J. Fleiszig

Supplementary Figure 1

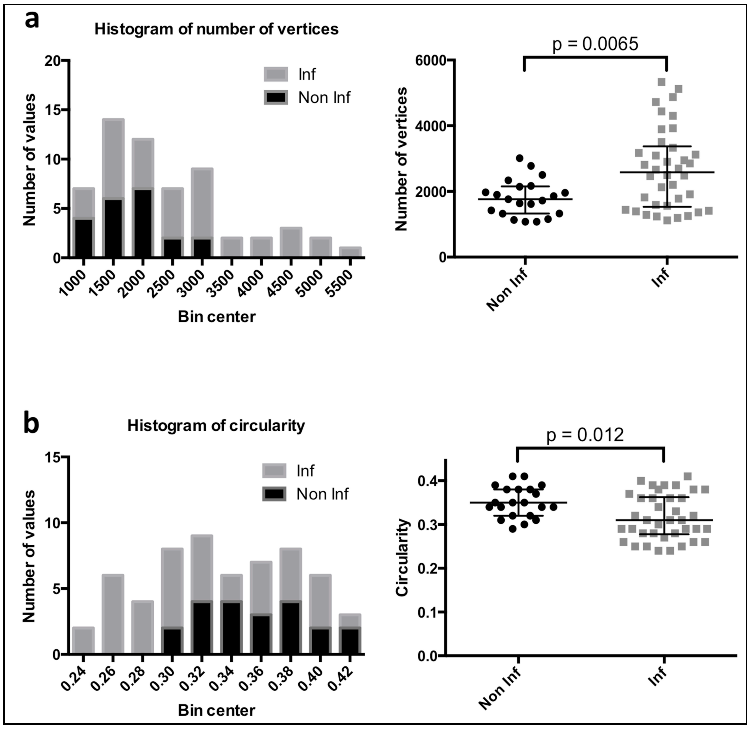

Fig. S1 Analysis of the distribution of CD11c+ cell shape changes in murine corneas before and after 4 h challenge with *P. aeruginosa* PAO1 *in vivo*. Control eyes were inoculated with PBS. (A) Distribution of the number of vertices per CD11c+ cell (range 1078 to 5334) (left). Median and interquartile ranges with points representing individual CD11c+ cells (right). (B) Distribution of CD11c+ cell circularity (range 0.24 to 0.41) (left). Median and interquartile ranges with points representing individual CD11c+ cells (right). P values were determined using the Mann-Whitney U test.

Supplementary Figure 2

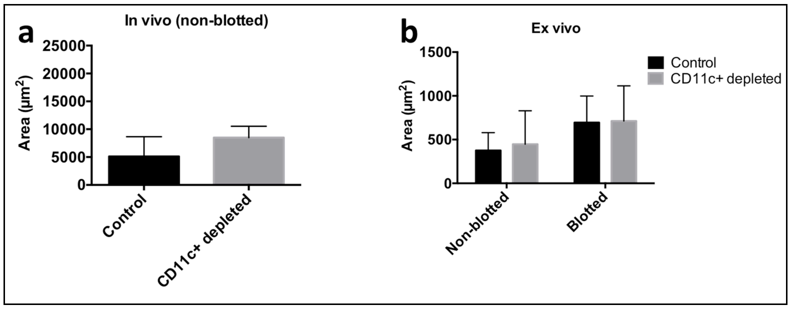

Fig. S2 (a) CD11c+ cell depletion did not affect *P. aeruginosa* PAO1 adhesion to healthy (non-blotted) murine corneas *in vivo* after 4 h exposure to bacteria. (b) CD11c+ cell depletion did not affect *P. aeruginosa* adhesion to healthy (non-blotted) or superficially-injured (blotted) corneas if experiments were performed *ex vivo* with 6 h exposure to bacteria.
